# Assortative mating on blood type: Evidence from one million Chinese pregnancies

**DOI:** 10.1073/pnas.2209643119

**Published:** 2022-12-14

**Authors:** Yao Hou, Ke Tang, Jingyuan Wang, Danxia Xie, Hanzhe Zhang

**Affiliations:** ^a^Institute of Economics, School of Social Sciences, Tsinghua University, Beijing, China 100084; ^b^Yanqi Lake Beijing Institute of Mathematical Sciences and Applications, Beijing, China 101407; ^c^School of Computer Science and Engineering, Beihang University, Beijing, China 100083; ^d^Pengcheng Laboratory, Shenzhen, China 518055; ^e^School of Economics and Management, Beihang University, Beijing, China 100083; ^f^Department of Economics, Michigan State University, East Lansing, MI 48824

**Keywords:** assortative mating, blood type, mate choice

## Abstract

Blood type is one of the most fundamental phenotypes in biological, medical, and psychological studies. Using a unique dataset of one million Chinese pregnancies, we find strong evidence from a group of statistical tests for assortative mating on blood type. After controlling for anthropometric and socioeconomic confounders, assortative mating remains robust.

## Significance

In the human population, spousal pairs have been found to share phenotypes, which demonstrates the highly nonrandom nature of human mate choice. However, assortative mating on blood type—one of the most fundamental phenotypes in biological, medical, and psychological studies—has not been investigated. Using a unique dataset from China, we provide statistical analysis to test whether matching on blood type is nonrandom and find a set of strong evidence for assortative mating on blood type. The findings are robust after we control for the effect of other possible mechanisms, and show that the spousal concordance on blood type we observe is attributable to not only an individual’s mate opportunity but also their mate choice.

Spousal pairs have been found to share a wide array of biological, socioeconomic, and psychological traits. One possible explanation is the highly nonrandom nature of human mate choice (1–12). Assortative mating may bring about important genetic consequences by increasing trait variance in a population, intensifying trait divergence, and providing lifesaving benefits; for example, during organ transplants (13–15). Motivated by significant multidisciplinary implications, studies reveal that many phenotypes, including body mass index (BMI), body height, and intelligence quotient (IQ), (10, 16–21) are highly correlated within spousal pairs. However, blood type—one of the most fundamental phenotypes—was discovered more than a century ago but remains untapped in studies on assortative mating (22). It is therefore of significance to investigate whether there is assortative mating on blood type in the population.

In this study, we aimed to investigate whether there is assortative mating on blood type and investigate possible sources of spousal concordance. To this end, we utilized a dataset obtained from 2014 to 2015 Chinese free prepregnancy checkup, a project that targets low-income married couples in urban areas and married couples in rural areas, who plan to get pregnant in the next 6 mo. With a full sample of 931,964 pregnant couples, the dataset represents an unbiased sample of the target population. Two dimensions of selection left unaccounted for are nonmarital fertility and marital nonfertility, but both are very uncommon in China: For example, the proportion of children conceived by the 1970 to 1979 birth cohort out of marriage is 0.3%, according to 2016 China Family Panel Studies. According to 2017 National Fertility Survey of China, the fraction of nonmarital fertility is 7.9%. The survey also shows that only 1.5% of married women aged between 15 and 60 had not got pregnant.

First, we performed Pearson’s chi-square test on blood type associations within spousal pairs to evaluate the degree of assortative mating on blood type. Our dataset represents an unbiased sample of the population. Second, we used a series of alternative measures to check whether the obtained results are robust. Third, we performed meta-analysis using subsamples divided according to the regions in which couples received prepregnancy checkups to control for the effect of population stratification on the estimation. Next, we performed logistic regression analysis and linear regression analysis to isolate our estimates from population stratification or the relationship-maintenance effect. Specifically, we regressed an individual’s blood type on spousal blood type and incorporated the share of blood type in the local population, share of the individual’s blood type in her ethnicity, and length of marriage in the regressions. Finally, we examined bivariate correlation between blood type and other phenotypes and performed mediation analysis to explore potential reasons for observed assortative mating on blood type.

## Results

We performed a series of statistical tests to explore assortative mating on blood type. We first used Pearson’s chi-square test on a contingency table for spousal pairs’ blood types to evaluate whether blood type influences human mate choice. Assortative mating typically refers to a mating choice pattern in which individuals with similar phenotypes mate with each other more frequently than the theoretical prediction under a random mating pattern. This definition implies that the degree of assortative mating on a certain phenotype can be measured by a chi-square test that compares the contingency table for pairs’ blood types with the contingency table generated by random matching. To perform this test, we used the full sample of Chinese prepregnancy checkup data and aggregated information on each spousal pair’s blood types into a contingency table. The raw dataset consists of 1,137,010 couples who were followed up and became pregnant within 6 mo after the prepregnancy exam, from all 31 provincial administrative regions of Mainland China. For convenience of our later analysis, we removed observations with incomplete information related to the couple’s blood types, living areas, birthplaces, ethnicity, and marital status. We obtained a sample of 931,964 couples with complete personal information to serve as our full sample. As the contingency table reports, diagonal elements have a higher frequency than expected by uniform random mating, which shows that spousal pairs with the same blood type are more likely to marry each other (Table 1). Pearson’s chi-square test on the full sample (chi-square statistic: 4020.942, *P*-value: 0.000, degree of freedom: 9, Cramer’s V: 0.038) further validates nonrandom mating.

**Table 1.** Observed mating pairs and ratios over expected mating pairs

		Female			
		A	B	AB	O
Male	A	88,168 +5.2%	77,223 +0.1%	24,426 +0.1%	86,224 −4.9%
	B	79,506 +2.2%	75,959 +6.1%	22,842 +0.9%	77,904 −7.4%
	AB	24,277 −1.8%	24,033 +5.7%	8,492 +18.1%	24,564 −8.1%
	O	91,135 −5.8%	83,310 −6.4%	26,584 −5.5%	117,317 +12.2%

*Note*: Percentages of observed mating pairs above the expected mating pairs are underlined.

We subsequently adopted alternative measures to evaluate the degree of assortative mating to investigate whether the conclusion obtained from the chi-square test is robust. The chi-square test shows whether we can reject the null hypothesis of random mating, but it cannot tell us the specific pattern the mating process follows if the null hypothesis is rejected. To learn whether mating choice is assortative, we are particularly interested in the diagonal elements of the contingency table. Specifically, we wanted to know whether the numbers for matching in these four specific cells are significantly different from expected under uniform random matching. To do this, we computed adjusted Pearson residuals that indicate whether the number of mating pairs in a specific cell is significantly different than expected and performed statistical tests on the statistics of diagonal elements at the α=5% level. It shows that the four pairs of blood types—i.e., the diagonal elements of the country-level contingency table, (A, A), (B, B), (AB, AB), and (O, O)—have adjusted Pearson residuals of 21.312, 22.420, 16.846, and 59.482, respectively, all of which are significantly higher than the two-sided critical value after Bonferroni correction, ±2.498. The results suggest that individuals of all blood types tend to marry those who share the same blood type.

We also used the Altham index, which is widely used in testing the association of unordered rows and columns of an r×s contingency table, to alternatively measure the overall extent of assortative mating (23–25). The Altham index uses the odds ratio of the likelihood of matching within different blood type pairs to capture the distance between the row–column associations in the observed contingency table and those generated by random matching patterns (see *Methods*). Its value is equal to zero if mating choice is random and increases with the degree of nonrandom row–column association. The estimate of the Altham index with the full sample is 2.063, which demonstrates a significant correlation in spousal blood types.

**Fig. 1.**
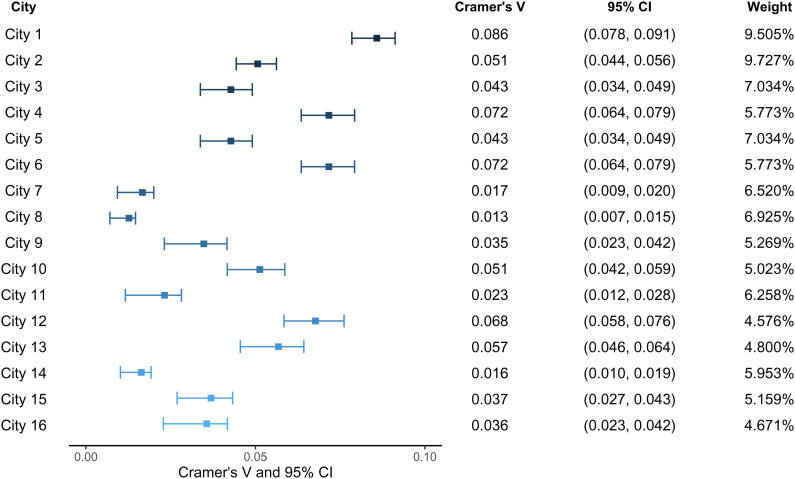
Forest plot of meta-analysis. The SE and 95% CI of Cramer’s V are estimated through bootstrap method with 1,000 replications. The weight assigned to each subsample is equal to the inverse of the error variance of the subsample’s Cramer’s V over the sum of the error variance’s inverses among subsamples. (see *Methods*).

Because of possible geographical heterogeneity of blood type distribution, we then restricted our analysis to local subsamples (couples who were born in the same region and received a prepregnancy checkup in this region) and performed a meta-analysis on assortative mating to investigate whether the assortative mating pattern still exists after controlling for possible subpopulation structure and whether this pattern is universal among different areas in China (15). Population stratification is one of the key drivers of spousal concordance that is independent of individuals’ mate choice. Individuals from subpopulations with residential proximity may naturally have more opportunities to mate. Moreover, they usually share similarities in their blood types because of their similar ethnic backgrounds. Without controlling for population stratification, we may overestimate the degree of assortative mating. Local subsample analysis is therefore proposed to relieve the estimation bias of assortative mating induced by population stratification. We identified locally matched observations by birthplace information and living area information provided by Chinese prepregnancy checkup data. Meta-analysis is carried out at city level (that is, we evaluate assortative mating within a city). We report statistical results for the 16 cities whose sample sizes (i.e., number of pregnant couples) are larger than 10,000 (Table 2 and Fig. 1). In 13 of the 16 cities, Pearson’s chi-square statistics indicate that mating choice is nonrandom at the 5% level of significance. Adjusted Pearson residuals further suggest that assortative mating on blood type is common in different areas, even after the subpopulation structure is controlled for. Blood-type pairs (O, O) typically have higher adjusted Pearson residuals than other blood type pairs, and thus show a higher degree of assortative mating. Also, the values of adjusted Pearson residuals we obtained from locally matched samples in each city are generally lower than those obtained from the national full sample. A similar pattern is observed in the forest plot, in which 12 of the 16 cities have an effect size, i.e., Cramer’s V, larger than 0.03 and seven among them have an effect size greater than 0.05 (Fig. 1). The fixed-effects estimate of Cramer’s V over the 16 subsamples is 0.046.

**Table 2.** Statistical tests at city level (16 cities with samples >10,000)

Sample size	Pearson’s chi-square statistic	Cramer’s V	Adjusted Pearson residuals			
			A, A	B, B	AB, AB	O, O
34,015	751.419**	0.086	16.367**	8.858**	5.768**	25.060**
29,955	230.625**	0.051	1.826	−5.149**	–0.441	10.506**
22,124	121.338**	0.043	0.250	−4.493**	1.758	6.147**
18,662	287.433**	0.072	7.720**	7.138**	0.212	10.398**
18,178	15.148	0.017	1.167	1.309	−0.385	3.716**
17,045	8.244	0.013	1.142	0.822	−0.286	−0.402
16,845	61.116**	0.035	2.321	2.878**	1.575	7.171**
16,668	131.567**	0.051	5.810**	2.487	4.539**	8.346**
16,284	26.275**	0.023	3.134**	0.844	1.471	3.041**
15,550	213.853**	0.068	−9.092**	−2.533**	0.787	5.925**
15,530	150.266**	0.057	−3.102**	−1.402	6.452**	−0.147
15,062	12.042	0.016	1.481	0.610	1.686	1.400
14,956	61.087**	0.037	−2.171	−3.215**	2.567**	3.299**
14,349	54.662**	0.036	2.003	4.212**	2.714**	5.527**
12,922	41.119**	0.033	4.558**	0.826	2.505**	4.362**
11,409	255.578**	0.086	−4.481**	−5.478**	1.079	10.643**

*Note*: ** Significant at the 5% level. The 5%-level critical value for Pearson’s chi-square test is 19.023 with a degree of freedom of nine. The 5%-level critical value for adjusted Pearson residuals with Bonferroni correction is 2.498 (see *Methods*).

Meta-analysis helps to eliminate the possible effects of subpopulation structure on assortative mating. However, another possible mechanism, relationship maintenance, may also confound estimation of assortative mating. The similarity of spousal pairs’ blood types could be explained by the concordance of living with a partner with the same blood type. As some studies have indicated, spousal pairs with similar phenotypes, such as alcohol consumption, could be more likely to remain in a relationship (26–28). If blood type is associated with some phenotypes, then couples with the same blood type might live in concord with each other because of the similarities they share and enroll in the prepregnancy checkup dataset, because their relationship has a lower probability of breaking up before they marry. Therefore, we perform regression analysis to isolate the effect of assortative mating on spousal concordance on blood type from that of relationship maintenance as well as that of subpopulation structure. The regressions will help us quantitatively evaluate how much variation in an individual’s blood type can be explained by assortative mating and the other two alternative mechanisms—population stratification and relationship maintenance. Specifically, we regress an individual’s blood type on their partner’s blood type and incorporate a group of control variables in the regression, which includes the share of the individual’s blood type in the population in their birthplace, the share in their ethnicity, the length of the marriage, and its interaction term with the partner’s blood type (see *Methods* for details). For comparison, we also run regressions without controlling for other factors.

The results are reported in Tables 3 and 4. In the odd-numbered columns, positive coefficients of indicator variables that show whether the individual and her partner have the same blood type offer evidence for assortative mating on blood type. After incorporating control variables, as reported in even-numbered columns, the magnitudes of indicator variables’ coefficients decline considerably, and most control variables show statistical significance; this validates that estimates of assortative mating can be biased by confounding factors such as population stratification and relationship maintenance. It can be further seen from the decline in estimates of indicator variables’ coefficients after incorporation of control variables that approximately 30 to 40% of spousal concordance on blood type observed in the odd-numbered columns in Tables 3 and 4 is attributed to confounders, while the remaining portion of spousal similarities can be ascribed to assortative mating. After incorporation of a group of control variables, the coefficients of the partner’s blood type are still statistically significant at the 1% level, which indicates highly nonrandom matching on blood type.

**Table 3.** Logistic regression analysis of the full sample: Wife’s mate choice

Variable	$I_{wife}^{A}$		$I_{wife}^{B}$		$I_{wife}^{AB}$		$I_{wife}^{O}$	
$I_{Husband}^{A}$	0.104*** (0.005)	0.076*** (0.006)						
$I_{Husband}^{B}$			0.115*** (0.005)	0.066*** (0.006)				
$I_{Husband}^{AB}$					0.203*** (0.012)	0.145*** (0.014)		
$I_{Husband}^{O}$							0.273*** (0.005)	0.197*** (0.005)
${BS}_{Wife}^{A}$		4.610*** (0.058)						
${BS}_{Wife}^{B}$				4.821*** (0.049)				
${BS}_{Wife}^{AB}$						12.054*** (0.206)		
${BS}_{Wife}^{O}$								4.238*** (0.035)
${EGS}_{Wife}^{A}$		1.804*** (0.176)						
${EGS}_{Wife}^{B}$				0.567* (0.316)				
${EGS}_{Wife}^{AB}$						4.821*** (0.603)		
${EGS}_{Wife}^{O}$								0.573*** (0.096)
Length of marriage		0.000 (0.000)		0.000** (0.000)		0.000 (0.000)		−0.000*** (0.000)
$I_{Husband}^{A}\times$Length of marriage		−0.000 (0.000)						
$I_{Husband}^{B}\times$Length of marriage				−0.000 (0.000)				
$I_{Husband}^{AB}\times$Length of marriage						0.000* (0.000)		
$I_{Husband}^{O}\times$Length of marriage								0.000** (0.000)
Observation	931,964	931,964	931,964	931,964	931,964	931,964	931,964	931,964

*Note*: Robust SEs are in parentheses. *Significant at 10% level, **5% level, ***1% level. $I_{Husband,i}^{g}$ and $I_{Wife,i}^{g}$ indicate whether husband and wife of couple i have type g blood, respectively. ${BS}_{Wife,i}^{g}$ and ${BS}_{Husband,i}^{g}$ are the share of type g blood individuals in the population of the birthplace of the wife or the husband in couple i. ${EGS}_{Wife,i}^{g}$ and ${EGS}_{Husband,i}^{g}$ represent the share of individuals with type g blood in the population of the ethnicity of the wife or the husband in couple i. ${Length\;of\;Marriage}_{i}$ (Unit: d) indicates the time gap between when couple i marries and when they receive a prepregnancy checkup.

**Table 4.** Logistic regression analysis of the full sample: Husband’s mate choice

Variable	$I_{Husband}^{A}$		$I_{Husband}^{B}$		$I_{Husband}^{AB}$		$I_{Husband}^{O}$	
$I_{Wife}^{A}$	0.104*** (0.005)	0.076*** (0.006)						
$I_{Wife}^{B}$			0.115*** (0.005)	0.066*** (0.006)				
$I_{Wife}^{AB}$					0.203*** (0.012)	0.146*** (0.014)		
$I_{Wife}^{O}$							0.273*** (0.005)	0.195*** (0.005)
${BS}_{Husband}^{A}$		4.667*** (0.055)						
${BS}_{Husband}^{B}$				4.898*** (0.047)				
${BS}_{Husband}^{AB}$						11.598*** (0.172)		
${BS}_{Husband}^{O}$								4.210*** (0.035)
${EGS}_{Husband}^{A}$		1.441*** (0.183)						
${EGS}_{Husband}^{B}$				0.772*** (0.269)				
${EGS}_{Husband}^{AB}$						5.005*** (0.623)		
${EGS}_{Husband}^{O}$								0.482*** (0.093)
Length of marriage		0.000 (0.000)		−0.000 (0.000)		−0.000* (0.000)		0.000 (0.000)
$I_{Wife}^{A}\times$ Length of marriage		−0.000 (0.000)						
$I_{Wife}^{B}\times$ Length of marriage				−0.000 (0.000)				
$I_{Wife}^{AB}\times$ Length of marriage						0.000* (0.000)		
$I_{Wife}^{O}\times$ Length of marriage								0.000** (0.000)
Observation	931,964	931,964	931,964	931,964	931,964	931,964	931,964	931,964

*Note*: Robust SEs are in parentheses. *Significant at 10% level, **5% level, ***1% level. $I_{Husband,i}^{g}$ and $I_{Wife,i}^{g}$ indicate whether husband and wife of couple i have type g blood, respectively. ${BS}_{Wife,i}^{g}$ and ${BS}_{Husband,i}^{g}$ are the share of type g blood individuals in the population of the birthplace of the wife or the husband in couple i. ${EGS}_{Wife,i}^{g}$ and ${EGS}_{Husband,i}^{g}$ represent the share of individuals with type g blood in the population of the ethnicity of the wife or the husband in couple i. ${Length\;of\;Marriage}_{i}$ (Unit: d) indicates the time gap between when couple i marries and when they receive a prepregnancy checkup.

The coefficients of variables with statistical significance represent the extent to which the corresponding mechanism can explain the individual’s blood type. By decomposing spousal concordance on blood type into several mechanisms, even-numbered columns in Tables 3 and 4 reflect the magnitude of different mechanisms’ effects on spousal similarities observed in data. The results show that both subpopulation structure and assortative mating play key roles in explaining the observed pattern, while relationship maintenance fails to be a convincing explanation. One’s blood type is mostly explained by the distribution of blood types in the population of her birthplace. A 1% rise in the share increases the odds ratio of having the corresponding blood type by approximately 4 to 5% for individuals with type A, B, or O blood and by approximately 12% for those with type-AB blood. In addition to local population structure, the individual’s ethnicity explains a considerable fraction of her blood type. A 1% increase in the share of her blood type in her ethnicity increases the odds ratio of having the corresponding blood type by approximately 1 to 2% for those with type A, B, or O blood and by approximately 5% for those with type-AB blood. Her partner’s blood type partner is also an effective predictor of her blood type, which provides evidence for assortative mating. If her partner has a given blood type, the odds ratio of having the same blood type will increase by approximately 8% for those with type-A blood, 7% for those with type-B blood, 15% for those with type-AB blood, and 20% for those with type-O blood (Tables 3 and 4). More assortative mating is observed within spousal pairs with type-AB blood or type-O blood. The almost equal estimates in Tables 3 and 4 suggest that the degrees of assortative mating are similar between females and males. We did not find strong evidence to support the argument that the similarity of spousal pairs’ blood types is associated with length of marriage, which is used as a proxy for relationship maintenance: The coefficients of the interaction terms of length of marriage and the indicator variables for partner’s blood type are insignificant or of small magnitude (lower than 0.001).

The estimated increase in the odds ratio roughly indicates the degree of assortative mating on blood type. To estimate the increase in the probability of matching between individuals that can be explicitly attributed to blood type assortative mating, we repeated the above regression analysis using a linear regression model and report the results in Tables 5 and 6. Like the results obtained by logistic regression analysis, individuals with the same blood type have a significantly higher probability of matching with each other than those with different blood types. The results are robust after controlling for a group of control variables. If one’s partner is of a specific blood type, the probability of having the same blood type will increase by approximately 1.6% for those with type A blood, 1.3% for those with type B blood, 1.2% for those with type AB blood, and 4.4% for those with type O blood (Tables 5 and 6). Again, linear regression analysis provides clear evidence to support assortative mating on blood type.

**Table 5.** Linear regression analysis of the full sample: Wife’s mate choice

Variable	$I_{wife}^{A}$		$I_{wife}^{B}$		$I_{wife}^{AB}$		$I_{wife}^{O}$	
$I_{Husband}^{A}$	0.022*** (0.001)	0.016*** (0.001)						
$I_{Husband}^{B}$			0.023*** (0.001)	0.013*** (0.001)				
$I_{Husband}^{AB}$					0.018*** (0.001)	0.012*** (0.001)		
$I_{Husband}^{O}$							0.061*** (0.001)	0.044*** (0.001)
${BS}_{Wife}^{A}$		0.961*** (0.012)						
${BS}_{Wife}^{B}$				0.984*** (0.010)				
${BS}_{Wife}^{AB}$						0.963*** (0.015)		
${BS}_{Wife}^{O}$								0.941*** (0.008)
${EGS}_{Wife}^{A}$		0.286*** (0.031)						
${EGS}_{Wife}^{B}$				0.114* (0.064)				
${EGS}_{Wife}^{AB}$						0.236*** (0.037)		
${EGS}_{Wife}^{O}$								0.167*** (0.023)
Length of marriage		0.000 (0.000)		0.000** (0.000)		0.000 (0.000)		−0.000*** (0.000)
$I_{Husband}^{A}\times$Length of marriage		−0.000 (0.000)						
$I_{Husband}^{B}\times$Length of marriage				−0.000 (0.000)				
$I_{Husband}^{AB}\times$Length of marriage						0.000** (0.000)		
$I_{Husband}^{O}\times$Length of marriage								0.000 (0.000)
Observation	931,964	931,964	931,964	931,964	931,964	931,964	931,964	931,964

*Note*: Robust SEs are in parentheses. *Significant at 10% level, **5% level, ***1% level. $I_{Husband,i}^{g}$ and $I_{Wife,i}^{g}$ indicate whether husband and wife of couple i have type g blood, respectively. ${BS}_{Wife,i}^{g}$ and ${BS}_{Husband,i}^{g}$ are the share of type g blood individuals in the population of the birthplace of the wife or the husband in couple i. ${EGS}_{Wife,i}^{g}$ and ${EGS}_{Husband,i}^{g}$ represent the share of individuals with type g blood in the population of the ethnicity of the wife or the husband in couple i. ${Length\;of\;Marriage}_{i}$ (Unit: d) indicates the time gap between when couple i marries and when they receive a prepregnancy checkup.

**Table 6.** Linear regression analysis of the full sample: Husband’s mate choice

Variable	$I_{Husband}^{A}$		$I_{Husband}^{B}$		$I_{Husband}^{AB}$		$I_{Husband}^{O}$	
$I_{Wife}^{A}$	0.022*** (0.001)	0.016*** (0.001)						
$I_{Wife}^{B}$			0.023*** (0.001)	0.013*** (0.001)				
$I_{Wife}^{AB}$					0.017*** (0.001)	0.012*** (0.001)		
$I_{Wife}^{O}$							0.062*** (0.001)	0.044*** (0.001)
${BS}_{Husband}^{A}$		0.970*** (0.011)						
${BS}_{Husband}^{B}$				0.986*** (0.009)				
${BS}_{Husband}^{AB}$						0.977*** (0.015)		
${BS}_{Husband}^{O}$								0.946*** (0.008)
${EGS}_{Husband}^{A}$		0.217*** (0.032)						
${EGS}_{Husband}^{B}$				0.144*** (0.054)				
${EGS}_{Husband}^{AB}$						0.181*** (0.037)		
${EGS}_{Husband}^{O}$								0.148*** (0.022)
Length of marriage		0.000 (0.000)		−0.000 (0.000)		−0.000*** (0.000)		0.000 (0.000)
$I_{Wife}^{A}\times$ Length of marriage		−0.000 (0.000)						
$I_{Wife}^{B}\times$ Length of marriage				−0.000 (0.000)				
$I_{Wife}^{AB}\times$ Length of marriage						0.000* (0.000)		
$I_{Wife}^{O}\times$ Length of marriage								0.000** (0.000)
Observation	931,964	931,964	931,964	931,964	931,964	931,964	931,964	931,964

*Note*: Robust SEs are in parentheses. *Significant at 10% level, **5% level, ***1% level. $I_{Husband,i}^{g}$ and $I_{Wife,i}^{g}$ indicate whether husband and wife of couple i have type g blood, respectively. ${BS}_{Wife,i}^{g}$ and ${BS}_{Husband,i}^{g}$ are the share of type g blood individuals in the population of the birthplace of the wife or the husband in couple i. ${EGS}_{Wife,i}^{g}$ and ${EGS}_{Husband,i}^{g}$ represent the share of individuals with type g blood in the population of the ethnicity of the wife or the husband in couple i. ${Length\;of\;Marriage}_{i}$ (Unit: d) indicates the time gap between when couple i marries and when they receive a prepregnancy checkup.

## Possible Reasons for Assortative Mating on Blood Type

**Fig. 2.**
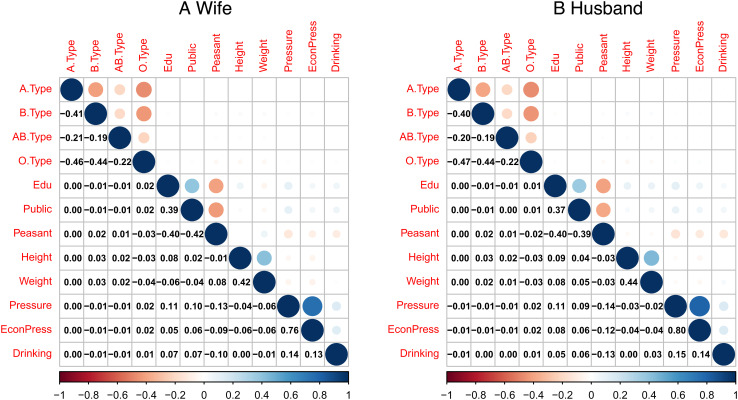
Bivariate correlation between blood type and other phenotypes. The variables “x.Type” indicate whether the individual has type x blood. Edu is a categorical variable that takes the value 1 if the individual receives no education, 2 if primary school education, 3 if middle school education, 4 if high school education, 5 if a bachelor’s degree, and 6 if postgraduate qualification. Public is a binary variable that indicates whether the individual works as a public employee. Peasant is a binary variable that represents if the individual is a peasant. Height (Unit: cm) and Weight (Unit: kg) record the individual’s height and weight when receiving the prepregnancy examination. Pressure is a categorical variable that documents the degree of pressure felt by the individual and takes the value 0 if they feel no pressure, 4 if the pressure is heavy, and a discrete value of 1, 2, or 3 if the degree of pressure is moderate. EconPress is a categorical variable that measures the individual’s economic pressure and is defined in a way that is similar to Pressure. Drinking is a categorical variable that rates how often the individual drinks and takes the value 0 if they never drink, 1 if they occasionally drink, and 2 if they often drink.

Having shown robust evidence for assortative mating on blood type, we investigate potential reasons. One possible explanation is that blood type may act as a proxy for other phenotypes. As previously stated, many studies have validated assortative mating on a group of phenotypes, such as BMI, weight, height, and IQ (10, 16–21). That is, individuals tend to choose a partner who shares similarities along these dimensions when making mate choices. If blood type is associated with these phenotypes, spousal concordance on blood type will be observed because of assortative mating. Using personal information provided by the dataset, we examine bivariate correlation between blood type and other phenotypes (Fig. 2). There appear to be some associations between blood type and the phenotypes we examine: education, job type, height, weight, pressure, and drinking habits. However, most associations have a relatively small correlation coefficient lying between −0.03 and 0.03.

To further explore to what extent assortative mating on blood type can be explained by its correlation with other phenotypes, we performed mediation analysis. Specifically, we first regressed the individual’s blood type on her partner’s using a logistic regression model, then incorporated a mediator—i.e., one of the partner’s phenotypes that might be associated with his blood type—to see whether and to what degree the effect of the partner’s blood type on the individual’s blood type is weakened after the mediator is included in the regression. We report the results of mediation analysis in Tables 7 and 8. As can be seen, the coefficients of the partner’s blood type decline after we included different mediators in the regression models, which shows that the associations between blood type and other phenotypes can explain assortative mating on blood type to a certain degree. We see from columns 2 to 9 in Table 7 and column 1 in Table 8 that the proportion of the coefficients of the partner’s blood type absorbed by mediators varies with blood type. For individuals with type B blood, when all mediators are included, the coefficients of the partner’s blood type are reduced by around 6 to 7%; for those with type A blood, the incorporation of mediators has little effect on estimation results for coefficients of the partner’s blood type, as shown in column 1 in Table 8. As for those with type AB blood or type O blood, the scale of mediator absorption is about 3 to 4%. However, a large fraction of assortativity remains unexplained. When we further included a group of control variables to isolate our measure of assortative mating from confounding factors—such as population stratification, province-level fixed effects, or even the individual’s phenotypes—in the regression, as indicated by the statistical significance of the coefficients of the partner’s blood type in columns 2 to 4 in Table 8, we still found strong evidence for assortative mating on blood type. These findings suggest that there could be other potential mechanisms for this pattern we observe in the data. Further investigation into this is left for future research.

**Table 7.** Mediation analysis with single mediators

Mediator	No mediators	Edu	Public	Peasant	Height	Weight	Pressure	EconPress	Drinking
$I_{Wife}^{A}$	0.104^***^ (0.005)	0.104^***^ (0.005)	0.104^***^ (0.005)	0.104^***^ (0.005)	0.104^***^ (0.005)	0.104^***^ (0.005)	0.104^***^ (0.005)	0.104^***^ (0.005)	0.104^***^ (0.005)
$I_{Wife}^{B}$	0.115^***^ (0.005)	0.114^***^ (0.005)	0.114^***^ (0.005)	0.113^***^ (0.005)	0.112^***^ (0.005)	0.113^***^ (0.005)	0.114^***^ (0.005)	0.114^***^ (0.005)	0.115^***^ (0.005)
$I_{Wife}^{AB}$	0.203^***^ (0.012)	0.203^***^ (0.012)	0.203^***^ (0.012)	0.202^***^ (0.012)	0.200^***^ (0.012)	0.201^***^ (0.012)	0.202^***^ (0.012)	0.203^***^ (0.012)	0.203^***^ (0.012)
$I_{Wife}^{O}$	0.273^***^ (0.005)	0.272^***^ (0.005)	0.273^***^ (0.005)	0.270^***^ (0.005)	0.269^***^ (0.005)	0.271^***^ (0.005)	0.272^***^ (0.005)	0.272^***^ (0.005)	0.273^***^ (0.005)
$I_{Husband}^{A}$	0.104^***^ (0.005)	0.104^***^ (0.005)	0.104^***^ (0.005)	0.104^***^ (0.005)	0.104^***^ (0.005)	0.104^***^ (0.005)	0.104^***^ (0.005)	0.104^***^ (0.005)	0.104^***^ (0.005)
$I_{Husband}^{B}$	0.115^***^ (0.005)	0.114^***^ (0.005)	0.114^***^ (0.005)	0.112^***^ (0.005)	0.111^***^ (0.005)	0.111^***^ (0.005)	0.114^***^ (0.005)	0.114^***^ (0.005)	0.115^***^ (0.005)
$I_{Husband}^{AB}$	0.203^***^ (0.012)	0.203^***^ (0.012)	0.203^***^ (0.012)	0.202^***^ (0.012)	0.200^***^ (0.012)	0.200^***^ (0.012)	0.202^***^ (0.012)	0.203^***^ (0.012)	0.203^***^ (0.012)
$I_{Husband}^{O}$	0.273^***^ (0.005)	0.273^***^ (0.005)	0.273^***^ (0.005)	0.270^***^ (0.005)	0.268^***^ (0.005)	0.268^***^ (0.005)	0.272^***^ (0.005)	0.272^***^ (0.005)	0.273^***^ (0.005)
Controls	No	No	No	No	No	No	No	No	No
Regional fixed effect	No	No	No	No	No	No	No	No	No
Individual phenotypes	No	No	No	No	No	No	No	No	No

*Note*: This table reports estimated coefficients of the partner’s blood type in logistic regressions under different model settings. Column 1 reports estimation results in baseline logistic models that regress the individual’s blood type on the partner’s blood type: $\begin{array}{c} \ln\left[p_{Wife,i}^{g}/\left(1-p_{Wife,i}^{g}\right)\right]=\beta_{0}+\beta_{1}I_{Husband,i}^{g}+\in_{i},g\in \left\{A,\;B,\;AB,\;O\right\} \end{array}$ and $\ln\left[p_{Husband,i}^{g}/\left(1-p_{Husband,i}^{g}\right)\right]=\beta_{0}+\beta_{1}I_{Wife,i}^{g}+\in_{i},\;g\in \left\{A,\;B,\;AB,\;O\right\}$, where $p_{Wife,i}^{g}$ and $p_{Husband,i}^{g}$ indicate the probability that the wife or the husband of couple i has type g blood. $I_{Husband,i}^{g}$ and $I_{Wife,i}^{g}$ refer to indicator variables that show whether the wife or the husband of couple i has type g blood. Columns 2 to 9 report results of logistic models that incorporate a mediator: $\ln\left[p_{Wife,i}^{g}/\left(1-p_{Wife,i}^{g}\right)\right]=\beta_{0}+\beta_{1}I_{Husband,i}^{g}+\beta_{2}{Mediator}_{Husband,i}+\in_{i},\;g\in \left\{A,\;B,\;AB,\;O\right\}$ and $\begin{array}{c} \ln\left[p_{Husband,i}^{g}/\left(1-p_{Husband,i}^{g}\right)\right]=\beta_{0}+\beta_{1}I_{Wife,i}^{g}+\beta_{2}{Mediator}_{Wife,i}+\in_{i},\;g\in \{A,\;B,\;AB,\;O\}, \end{array}$ where ${Mediator}_{Husband,i}$ and ${Mediator}_{Wife,i}$ measure the phenotype of the wife or the husband of couple i.

**Table 8.** Mediation analysis with all mediators

Mediator	All mediators	All mediators	All mediators	All mediators
$I_{Wife}^{A}$	0.104^***^ (0.005)	0.076^***^ (0.006)	0.076^***^ (0.006)	0.076^***^ (0.006)
$I_{Wife}^{B}$	0.108^***^ (0.005)	0.066^***^ (0.006)	0.066^***^ (0.006)	0.066^***^ (0.006)
$I_{Wife}^{AB}$	0.197^***^ (0.012)	0.145^***^ (0.014)	0.144^***^ (0.014)	0.144^***^ (0.014)
$I_{Wife}^{O}$	0.264^***^ (0.005)	0.197^***^ (0.005)	0.198^***^ (0.005)	0.198^***^ (0.005)
$I_{Husband}^{A}$	0.104^***^ (0.005)	0.076^***^ (0.006)	0.076^***^ (0.006)	0.076^***^ (0.006)
$I_{Husband}^{B}$	0.107^***^ (0.005)	0.066^***^ (0.006)	0.066^***^ (0.006)	0.066^***^ (0.006)
$I_{Husband}^{AB}$	0.196^***^ (0.012)	0.146^***^ (0.014)	0.144^***^ (0.014)	0.144^***^ (0.014)
$I_{Husband}^{O}$	0.263^***^ (0.005)	0.195^***^ (0.005)	0.196^***^ (0.005)	0.196^***^ (0.005)
Controls	No	Yes	Yes	Yes
Regional fixed effect	No	No	Yes	Yes
Individual phenotypes	No	No	No	Yes

*Note*: This table reports estimated coefficients of the partner’s blood type in logistic regressions under different model settings. Column 1 reports results of the logistic model that incorporates all mediators—i.e., the partner’s phenotypes examined in columns 2 to 9 of Table 7. Column 2 reports results of the logistic model that incorporates all mediators examined in columns 2 to 9 of Table 7 and additional control variables, including the share of the individual’s blood type in the population living in her birthplace, the share in their ethnicity, length of marriage, and its interaction term with the partner’s blood type. Column 3 shows results of logistic models with incorporation of all mediators examined in column 2 to 9 of Table 7, control variables specified in the model used in column 2 as well as provincial-level fixed effects. Finally, column 4 adopts a logistic model that adds the individual’s phenotypes that are examined in column 2 to 9 of Table 7, into the model used in column 3 (see *Methods* for details).

**Table 9.** List of variables used in analysis

Description	Value
Blood type	A, B, AB, O
Province the respondent currently lives in	province index
City the respondent currently lives in	city index
Province the respondent was born in	province index
City the respondent was born in	city index
Ethnic group	ethnic index
Duration between marriage registration and prepregnancy exam	Length (days)
Respondent's education level	1 to 6
Whether the respondent works as a public employee	binary
Whether the respondent is a peasant	binary
Respondent's height	in cm
Respondent's weight	in kg
The degree of pressure felt	0 to 4
The degree of economic pressure felt	0 to 4
How often the respondent drinks	0 to 2

## Conclusion

In summary, we provide evidence of assortative mating on blood type. The degree of assortative mating varies among individuals with different blood types and across locations. Our findings are robust after we control for other possible mechanisms, such as environmental confounding and relationship maintenance. We further examine potential mechanisms for these observations

Our study makes two contributions. First, our empirical results shed light on nonrandom matching on blood type—one of the most well-known human phenotypes—which, to the best of our knowledge, has not been fully investigated. Assortative mating on blood type will have important genetic consequences by influencing the direction of the evolution of blood type distribution in the population. On the one hand, it will intensify trait divergence compared with random mating, and thus heighten the population’s response to directional natural selection (29, 30). On the other hand, it reduces the heterozygosity of the population’s blood type and may promote inbreeding depression (31). These evolutionary effects render it important to investigate this issue.

Second, we improve the causal inference of assortative mating using a group of approaches. To mitigate the estimation bias caused by population stratification, we restrict our analysis to locally matched subsamples to perform meta-analysis. We further address this concern by running regressions with control variables. Other possible mechanisms are also controlled for in our analysis. Our robust results show causal evidence for assortative mating on blood type.

We acknowledge the limitations of our study. First, couples who are lost to follow-up or fail to become pregnant after the prepregnancy examination are missing from our dataset. Selection into the sample introduced by the two kinds of prescreening could be (but does not appear to be) a potential source of bias. Second, although overall evidence for assortative mating was found and some potential mechanisms behind the pattern were examined, a large fraction of the assortative mating we observe from the data remains unexplained. It would be of interest to understand the underlying mechanisms in future research. Third, we should be cautious when extrapolating findings in the Chinese population to other populations. Further evidence is needed to investigate whether the nonrandom matching pattern of blood types is also robust in other populations.

**Fig. 3.**
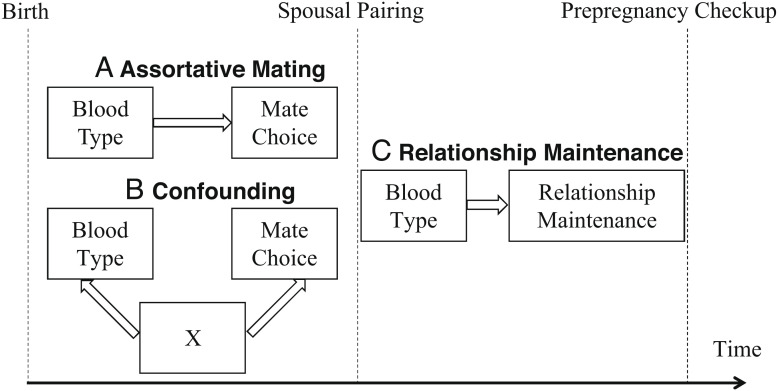
Possible mechanisms of spousal concordance on blood type. Mechanism *A* (Assortative mating) refers to the process whereby partners choose each other based on their similarity in blood type. Mechanism *B* (Confounding) indicates confounding factors that may influence both blood type and mate choice; population stratification is the most common confounding factor. Mechanism *C* (Relationship Maintenance) reflects the possibility that spousal pairs with the same blood type are more likely to maintain a long-term relationship and be enrolled in the observed sample.

Finally, we sought to avoid our estimates of the degree of assortative mating from being confounded by other factors (6, 21, 28, 32–42), such as population stratification and relationship maintenance after spousal pairing. We acknowledge that it is difficult to infer a causal relationship between blood type similarity and mate choice. As previously noted, when a latent subpopulation structure underlies the observed sample, the positive association of blood type within spousal pairs can be attributed to systematic differences among subpopulations that arise from the ancestral differences they inherit. Under this circumstance, assortative mating will thus be overestimated if homogeneous sampling is assumed. An alternative explanation for the similarity of blood types within spousal pairs is that blood type concordance helps spousal pairs maintain their relationship. Partners with the same blood type might share similar phenotypes, and thus find it easier to get along and maintain their relationship, which increases their probability of being enrolled in the study and leads to biased estimation. All three possible mechanisms may result in spousal concordance on blood type (Fig. 3). We strove to control for confounding factors by incorporating a group of control variables to indicate the blood type distribution of subpopulations and the length of couples’ relationship when estimating the extent of assortative mating on blood type using regression models. However, there might still be concern regarding whether confounding factors have been effectively ruled out and a causal relationship between blood type concordance and mate choice is clearly identified. To further study assortative mating on blood type in future research, we will need to carefully distinguish this mechanism from the other two possible explanations.

## Methods

### Data.

#### Chinese prepregnancy checkup data.

In this study, we used 2014 to 2015 Chinese prepregnancy checkup data to perform statistical analysis. Our dataset is from the National Free Preconception Health Examination Project (NFPHEP), which was launched by the National Health Commission (NHC) and the Ministry of Finance of the People’s Republic of China and offers free prepregnancy examinations for low-income married couples in urban areas and married couples in rural areas who plan to get pregnant in the next 6 mo. The nationwide project covers married couples with pregnancy plans in 2,907 counties from all 31 provincial administrative regions of Mainland China. Aiming to reduce birth defects and improve the health of newborns, China’s NHC strives to expand coverage of the survey with the aid of local communities and family planning service agencies. According to China’s NHC, the survey covered over 95% of the targeted population between 2014 and 2015 (43, 44). Couples who had made plans for pregnancy were enrolled by local community staff and given prepregnancy examinations. Blood samples are collected during the examination and sent to local laboratories for blood type testing. In addition to the results of physical examinations, the NFPHEP also collects all participants’ basic personal information—e.g., sex, address of residence, ethnicity, birthplace, and marriage—via a standardized questionnaire as well as participants’ identification cards (45–47). Two follow-up telephone interviews are conducted after the examination by trained nurses. The first is performed within 3 mo after the examination to check pregnancy status and the second is carried out within 1 y after the first follow-up interview to track the pregnancy outcome. After excluding couples who are lost to follow-up or fail to become pregnant after the prepregnancy examination, 1,137,010 couples are included in the dataset. Informed consent is signed by all project participants before enrollment. This study was approved by NHC.

#### Subsample with complete personal information.

We removed observations with incomplete information related to the couple’s blood types, living areas, birthplaces, ethnicity, marriage, education, job type, body height, weight, pressure, and drinking habits, which we used in our statistical analysis, and obtained a sample of 931,964 couples for the full sample of our study. The raw dataset of Chinese prepregnancy checkup data was filtered in Stata.

### Statistical Analysis.

#### Contingency table.

We broke down the numbers of matches between individuals with different blood types by sex to produce a 4×4 contingency table, with the number in grid (i, j) $N_{i,j}$ representing the frequency of matching between males with type i blood and females with type j blood. The ratios of observed frequencies over expected frequencies are also reported, with gird (i, j) computed as $N_{i,j}/E_{i,j}=N_{i,j}/\left(R_{i}\times C_{j}/N\right)$, where $R_{i}$ and $C_{j}$ denote row and column marginal totals and N represents the grand total. The contingency table was produced using R.

#### Pearson’s chi-square test.

We performed a chi-square test on a contingency table of blood types obtained from the full sample to test the association between an individual’s blood type and her partner’s blood type, with a null hypothesis that matching behavior is independent of spousal pairs’ blood types. The 5%-level critical value for Pearson’s chi-square test is 19.023 with a degree of freedom of nine. Cramer’s V was also reported to measure the effect size for the chi-square test. Pearson’s chi-square test was performed using R.

#### Adjusted Pearson residuals.

The adjusted Pearson residual of grid (i, j) is computed as

[1] $${\tilde{r}}_{i,j}=\frac{N_{i,j}-E_{i,j}}{\sqrt{E_{i,j}\left(1-R_{i}/N\right)\left(1-C_{j}/N\right)}}.$$

It follows a standard normal distribution N(0,1) in the null hypothesis of random matching, which enables us to perform statistical tests on nonrandom mating. The 5%-level critical value for adjusted Pearson residuals is ±2.498. It is corrected by Bonferroni correction. To test the four diagonal elements in the contingency table, the new alpha level should be set as $\alpha_{Bon}=\frac{5\%}{4}=1.25\;\%$ and the corresponding critical value ${N(0,1)}_{1-\alpha_{Bon}/2}=2.498$. Computation of adjusted Pearson residuals and related statistical tests were performed using R.

#### The Altham index.

We used the Altham index as an alternative measure of assortative mating, which can be written as

[2] $$\begin{array}{c} {\left[\sum_{i\in G}\sum_{j\in G}\sum_{k\in G}\sum_{l\in G}{\left|\log\left(\frac{P\left(I_{\mathrm{Wife}}^{i}=1,I_{\mathrm{Husband}}^{j}=1\right)P\left(I_{\mathrm{Wife}}^{k}=1,I_{\mathrm{Husband}}^{l}=1\right)}{P\left(I_{\mathrm{Wife}}^{i}=1,I_{\mathrm{Husband}}^{l}=1\right)P\left(I_{\mathrm{Wife}}^{k}=1,I_{\mathrm{Husband}}^{j}=1\right)}\right)\right|}^{2}\right]}^{1/2},\;G=\left\{A,,,\;,B,,,\;,A,B,,,\;,O\right\}, \end{array}$$

where $I_{Husband}^{g}$ and $I_{Wife}^{g}$ refer to indicator variables that show whether the wife or the husband has type g blood, and $P\left(I_{\mathrm{Wife}}^{g}=1,\;I_{\mathrm{Husband}}^{h}=1\right)$ represents the probability of matching between females with type g blood and males with type h blood, which is estimated by the share of this type of matching in the full sample. The higher the index, the more nonrandom the mate choice; it is equal to zero when matching is random. The Altham index was computed using R.

#### Meta-analysis.

As a sensitivity analysis, we restricted samples for Pearson’s chi-square test to locally matched couples to prevent our test on assortative mating from being confounded by population stratification. Specifically, we covered couples who were born in the same birthplace and received prepregnancy checkups in this area and stratify them by their birthplace. Meta-analysis is performed at the city level, which allows more granular segmentation for 16 locally matched subsamples. The meta-analysis results of chi-square test and effect size (Cramer’s V) estimation among subsamples are reported through a summary table and a forest plot. The SE and 95% CI of Cramer’s V are estimated through a bootstrap method with 1,000 replications. A fixed-effects meta-analysis model is further utilized to estimate the average true effect size of assortative mating on blood type over the 16 cities $\theta_{pop}$. The model estimates it by computing the weighted average of true city-specific effect sizes $\theta_{j}\;\left(j=1,2,...,16\right)$ that have been estimated y the proceeding meta-analysis,

$$\theta_{pop}=Ave\left(\theta_{j}\right)=\sum_{j=1}^{16}W_{j}\theta_{j},$$

in which the weight assigned to each subsample $W_{j}$ is equal to the inverse of the error variance of the subsample’s Cramer’s V over the sum of the error variance’s inverses among subsamples. The full sample was filtered in Stata to obtain locally matched subsamples. The bootstrap estimation of the SE and 95% CI of Cramer’s V among subsamples was also processed in Stata. The rest parts of meta-analysis of subsamples were performed using R.

#### Regression analysis.

We investigated the degree of assortative mating on blood type with logistic regression models. The models are specified as Eqs. **3** to **6**, where $p_{Wife,i}^{g}$ and $p_{Husband,i}^{g}$ indicate the probability that the wife or the husband of couple i has type g blood. $I_{Husband,i}^{g}$ and $I_{Wife,i}^{g}$ refer to indicator variables that show whether the wife or the husband of couple i has type g blood. ${BS}_{Wife,i}^{g}$ and ${BS}_{Husband,i}^{g}$  suggest the share of individuals with type g blood in the population of the birthplace of the wife or the husband in couple i, which is estimated by the population in this city covered in our full sample. ${EGS}_{Wife,i}^{g}$ and ${EGS}_{Husband,i}^{g}$  represent the share of individuals with type g blood in the population of the ethnicity of the wife or the husband in couple i, which is estimated by the population in this ethnicity covered in our full sample. Finally, ${Length\;of\;Marriage}_{i}$ indicates the time duration between when couple i get married and when they receive a prepregnancy checkup. $I_{Wife,i}^{g}\times {Length\;of\;Marriage}_{i}$ and $I_{Husband,i}^{g}\times {Length\;of\;Marriage}_{i}$ are interaction terms of the indicator variables and the length of marriage of this couple. We also repeated the above steps using linear regression model, to explicitly estimate the increase in probability of matching induced by assortative mating on blood type.

[3] $$\begin{array}{c} \ln\left(\frac{p_{Wife,i}^{g}}{1-p_{Wife,i}^{g}}\right)=\beta_{0}+\beta_{1}I_{Husband,i}^{g},\;\;\;\;\;\;\;\;g\in \left\{A,\;B,\;\mathrm{AB},\;O\right\}. \end{array}$$

[4] $$\begin{array}{cc} \ln\left(\frac{p_{Wife,i}^{g}}{1-p_{Wife,i}^{g}}\right)= & \beta_{0}+\beta_{1}I_{Husband,i}^{g}+\beta_{2}{BS}_{Wife,i}^{g}\\ & +\beta_{3}{EGS}_{Wife,i}^{g}+\beta_{4}{Length\;of\;Marriage}_{i}\\ & +\beta_{5}I_{Husband,i}^{g}\times {Length\;of\;Marriage}_{i},\;\\ & g\in \{A,\;B,\;AB,\;O\}. \end{array}$$

[5] $$\begin{array}{c} \ln\left(\frac{p_{Husband,i}^{g}}{1-p_{Husband,i}^{g}}\right)=\beta_{0}+\beta_{1}I_{Wife,i}^{g},\;g\in \left\{A,\;B,\;\mathrm{AB},\;O\right\}. \end{array}$$

[6] $$\begin{array}{cc} \ln\left(\frac{p_{Husband,i}^{g}}{1-p_{Husband,i}^{g}}\right)= & \beta_{0}+\beta_{1}I_{Wife,i}^{g}+\beta_{2}{BS}_{Husband,i}^{g}\\ & +\beta_{3}{EGS}_{Husband,i}^{g}+\beta_{4}{Length\;of\;Marriage}_{i}\\ & +\beta_{5}I_{Wife,i}^{g}\times {Length\;of\;Marriage}_{i},\;\\ & g\in \left\{A,\;B,\;\mathrm{AB},\;O\right\}. \end{array}$$

Besides the examination of the degree of assortative mating via logistic regression models, we also performed mediation analysis to explore potential mechanisms behind the observed pattern. Specifically, we included different mediators the regression models in Eqs. **3** and **5** and estimated the decline of the coefficients of the partner’s blood type that can be attributed to the incorporation of mediators, which is shown as Eqs. **7** and **8**.

[7] $$\begin{array}{c} \ln\left(\frac{p_{Wife,i}^{g}}{1-p_{Wife,i}^{g}}\right)=\beta_{0}+\beta_{1}I_{Husband,i}^{g}+\beta_{2}{Mediator}_{Husband,i},\;\\ \;g\in \left\{A,\;B,\;\mathrm{AB},\;O\right\}. \end{array}$$

[8] $$\begin{array}{c} \ln\left(\frac{p_{Husband,i}^{g}}{1-p_{Husband,i}^{g}}\right)=\beta_{0}+\beta_{1}I_{Wife,i}^{g}+\beta_{2}{Mediator}_{Wife,i},\;\\ \;g\in \left\{A,\;B,\;\mathrm{AB},\;O\right\}, \end{array}$$

where ${Mediator}_{Wife,i}$ and ${Mediator}_{Husband,i}$ measure the phenotype of the wife and the husband of couple i, respectively. The mediators we employed are education, whether the individual is a public employee, whether they work as a peasant, their height and weight, the pressure they feel, the economic pressure they feel, and their drinking habit. For all models, we adopted robust SEs in the model estimation to ensure that our estimates are robust when the model specification is incorrect. Regression analysis was performed using Stata.

## Data Availability

We used 2014 to 2015 Chinese prepregnancy checkup data available from the Institute of Science and Technology of the NHC of the People’s Republic of China. Data and code have been deposited in https://cloud.tsinghua.edu.cn/d/e86e227d8e66475ba790/ (48).
